# Ruptured ileocolic artery pseudoaneurysm: case report

**DOI:** 10.1590/1677-5449.210163

**Published:** 2022-01-07

**Authors:** Carolina Vasconcellos Sant’Anna, Felipe Garcia Kuhl, Alane Miranda Leite, Selma Regina de Oliveira Raymundo, André Rodrigo Miquelin, Vitória Acar, Vitor Brumato Fachini, Matheus Rafael Canuti

**Affiliations:** 1 Faculdade de Medicina de São José do Rio Preto – FAMERP, São José do Rio Preto, SP, Brasil.

**Keywords:** endovascular procedure, pseudoaneurysm, superior mesenteric artery

## Abstract

Visceral artery aneurysms (VAAs) and visceral artery pseudoaneurysms (VAPAs) are rare conditions and are potentially lethal when they rupture. They are usually found as incidental findings on computed tomography (CT) scans of asymptomatic patients. Although conventional open surgery is currently considered the gold standard treatment, the endovascular approach has gained prominence as a minimally invasive procedure with lower surgical risk. In this approach, use of coil embolization in saccular VAAs and VAPAs and implantation of flow-modulating stents constitute alternative treatments for fusiform aneurysms. We present the case of a 51-year-old female patient complaining of acute abdominal pain, tachycardia, and hypotension, with evidence of abdominal bleeding on CT angiography, who was diagnosed with a ruptured ileocolic artery (ICA) pseudoaneurysm. She underwent early endovascular treatment for ICA embolization, which was successful and achieved clinical improvement.

## INTRODUCTION

Visceral artery aneurysms (VAAs) and visceral artery pseudoaneurysms (VAPAs) are rare, with incidence in the range of 0.01 to 0.2%.^1^ Technical developments in imaging exams for detecting intra-abdominal diseases, such as abdominal computed tomography angiography (angio-CT) and Doppler ultrasound (USD),^2^^,^^3^ and the increased frequency of manipulation of the biliary tract have led to increases in prevalence rates and incidental findings of aneurysms. While VAAs are primarily caused by hypertension, atherosclerosis, dysplasia, and connective tissue diseases, VAPAs are associated with traumas, iatrogenic injuries, and inflammation or infection.^4^ The splenic artery is the most often involved in aneurysms and pseudoaneurysms, followed by the hepatic artery, celiac trunk, and inferior mesenteric artery.^5^

Compared with true aneurysms, VAPAs more frequently involve the gastroduodenal and superior mesenteric arteries (SMA) and ruptured pseudoaneurysms of the SMA are associated with a 37% mortality rate.^6^

Aneurysms and pseudoaneurysms of the ileocolic arteries (ICA) are rare and generally asymptomatic, but when symptomatic they are associated with signs of rupture such as abdominal pains, lower intestinal bleeding, and intra-abdominal hemorrhage, or signs of hypovolemic shock.^7^ Conventional surgery is considered the gold standard treatment, but endovascular treatment (ET) is gaining acceptance, particularly for management of patients at high risk from surgery.^8^^,^^9^

We present the case of a female patient diagnosed with a ruptured VAPA of the ICA who underwent successful ET. We highlight the importance of knowledge of this condition, which, while rare and potentially fatal, can also have a favorable outcome if diagnosed and treated early.

This study was approved by the Research Ethics Committee at our institution (CAAE: 50239621.8.0000.5415, consolidated opinion number: 4.919.534).

## DESCRIPTION OF THE CASE

The patient was a 51-year-old female admitted via emergency because of intense upper abdominal pains with onset 2 days previously, progressive worsening, hour by hour, and no response to analgesics. At admission, her general condition was regular, with cutaneous-mucosal pallor, cold skin, blood pressure of 125 x 59 mmHg, heart rate of 116 bpm, respiratory rate of 17 breaths/min, and body temperature of 36.1º. On physical examination, her abdomen was flat, with mild pain on deep palpation of the superior region, no masses, and no pain on decompression. Abdominal angio-CT was ordered, showing a large intraperitoneal hematoma in the mesogastric region measuring approximately 800 cm^3^ and presence of a moderate volume of free liquid in the abdominal cavity. The SMA was patent and a fusiform dilatation of the ICA was visible, with irregular outlines and contrast leakage to the right throughout its course (Figures 11B). The patient reported no history of prior disease and no continuous medication usage, but said she had undergone videolaparoscopic cholecystectomy several years previously.

**Figure 1:**
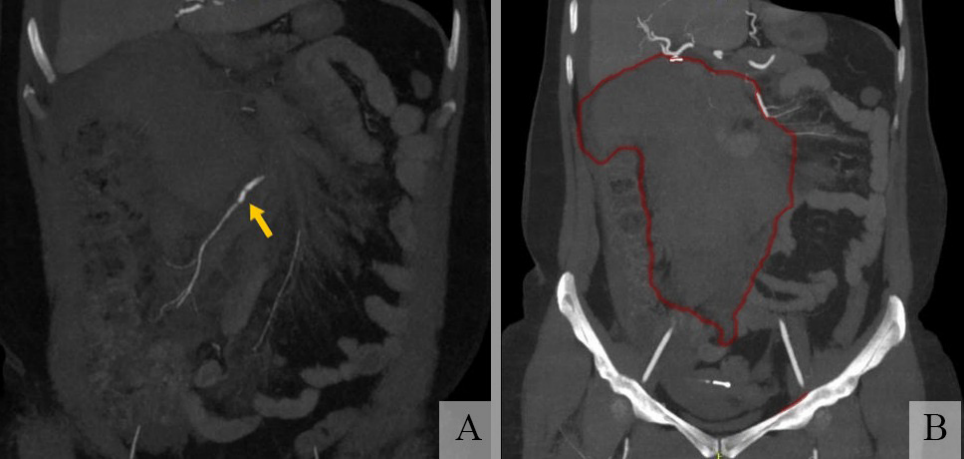
Abdominal angiotomography showing: **(A)** superior mesenteric artery with fusiform dilatation of the ileocolic artery, which has irregular borders and contrast leakage; **(B)** large intraperitoneal mesenteric hematoma and presence of free liquid in the abdominal cavity.

The patient was transferred to the catheterization laboratory and underwent arteriography, using the Seldinger technique to fit a 5Fr Performa^®^ hemostasis valve (Merit Medical Systems, Inc., Utah, United States) and position a Cobra Performa^®^ catheter (Merit Medical Systems, Inc., Utah, United States), followed by injection of contrast, which showed the dilatation and contrast leakage from the ICA **(**Figures 22B). Selective catheterization of the ICA was performed with a Progreat microcatheter (Terumo Corporation, Tokyo, Japan) and the decision was taken to embolize this artery by releasing Axium Prime™ 2.5 x 6 cm microcoils (Medtronic, Dublin, Ireland) into the area at the termination of the dilatation and Axium™ 2.5 x 8 cm microcoils (Medtronic, Dublin, Ireland) to seal the outflow, followed by release of Nester^®^ embolization coils (Cook Medical Inc., Indiana, United States) into the contrast leakage area (five 14 x 20 mm coils and two 14 x 18 mm coils), sealing the inflow (Figure 3). After the procedure, the patient reported improvement in pain and was hemodynamically stable. She was discharged from hospital on the 3rd postoperative day. At a 3-month follow-up, she was asymptomatic and control angio-CT showed no leakage or retroperitoneal hematoma (Figures 44B).

**Figure 2:**
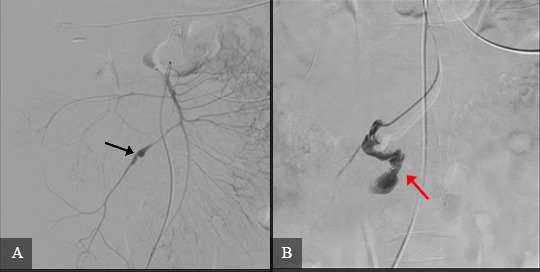
Angiography of the abdominal arteries showing: **(A)** fusiform pseudoaneurysm in the mid ileocolic artery (black arrow); **(B)** contrast leakage, indicative of rupture (red arrow).

**Figure 3:**
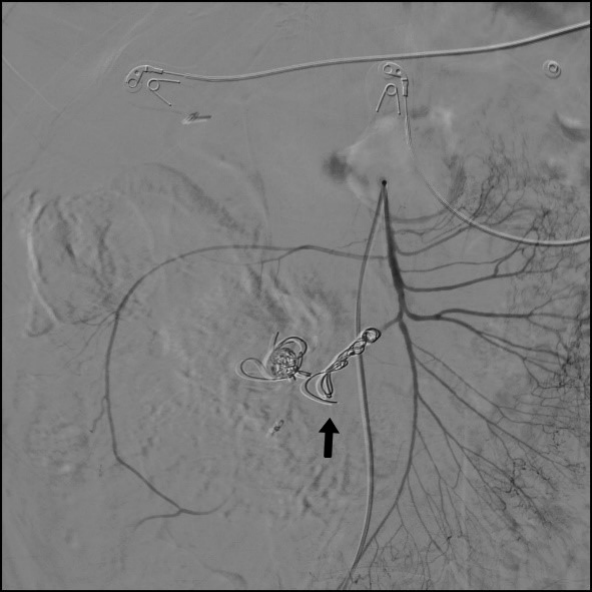
Control angiography after embolization of the ileocolic artery with microcoils, showing outflow and inflow sealed and no leakage (black arrow).

**Figure 4:**
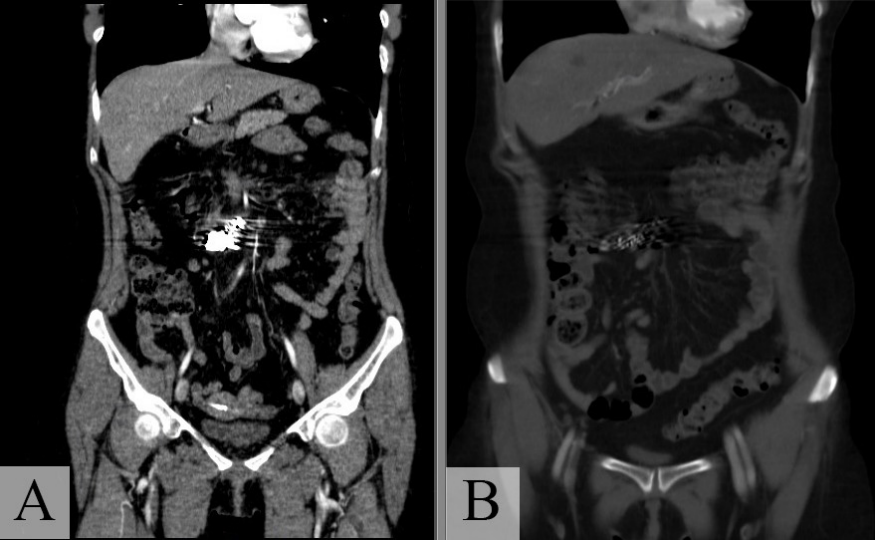
Control angiotomography 3 months after the procedure showing: **(A)** the microcoils positioned in the ileocolic artery and no leakage; **(B)** image of the entire abdomen, free from retroperitoneal hematoma.

## DISCUSSION

VAAs and VAPAs are rare manifestations, with incidence in the range of 0.1 to 2%,^1^ but, despite their low incidence, they have a high rate of rupture (25%) with significant morbidity and mortality (70%).^5^^,^^10^ They are caused by a variety of etiologies and initial diagnosis can be achieved with USD and computed tomography (CT) with sensitivity of 50% and 67%, respectively. Angiography is the gold standard, because it enables more specific identification of location and confirmation of collateral flow and can also be used for treatment.^11^ VAPAs of the SMA are most frequently caused by inflammation due to pancreatitis or other etiologies, but can also be caused by iatrogenic traumas^6^ during surgery or angiography or by accidents or penetrating traumas.^11^ The increased frequency of manipulation of the biliary tract with percutaneous endoscopic techniques and catheterization for intravascular chemoembolization have led to increased incidence of pseudoaneurysmal degeneration of visceral vessels, in addition to the role played by arterial trauma after laparoscopic treatment of intraperitoneal and retroperitoneal diseases.^12^

With the dissemination of imaging techniques such as angio-CT and magnetic resonance angiography, diagnoses of VAAs and VAPAs have increased, very often as incidental findings during examinations ordered for other abdominal diseases.^10^ The clinical presentation of these lesions is often vague and there are no clear signs to warn of imminent rupture. It is recommended that any suspected VAPA should be diagnosed and managed rapidly because of the high rates of rupture and hemorrhage.^11^ Pitton et al.^13^ found that VAPAs are more likely to rupture than true aneurysms (76.3% vs. 3.1%, respectively).

It was therefore necessary to classify the lesion in order to decide whether to perform a procedure surgical (open or catheter embolization) or adopt conservative follow-up.^14^ Conventional open techniques include resection with revascularization and ligature or final resection of the organ (generally splenectomy) and remain the gold standard treatment.^8^^,^^15^ However, they can very often cut off the blood flow in the principal artery and cause intestinal ischemia of varying degrees.^14^

Tulsyan et al.^12^ demonstrated that conventional surgery can increase morbidity and mortality in cases with difficult to access sites, with technical difficulties, or with associated comorbidities that prevent vascular reconstruction, and in patients admitted in critical conditions (acute bleeding and rupture). They observed a 96% success rate for endovascular coil embolization.^12^

Certain limitations of open surgery make minimally invasive endovascular procedures an option for patients who are at greater risk from surgery.^8^^,^^9^^,^^16^ For hemodynamically stable patients, ET remains the first-choice option for known arterial bleeding and is considered safe and effective for treating VAPAs. One advantage of this type of approach is that it enables treatment to be tailored to different conditions, making the method more precise for reducing postoperative morbidity. Complications include rupture of the sac, ischemia distal of the pseudoaneurysm sac, and the possibility of arterial flow into the pseudoaneurysm if restoration of flow proves time-consuming (post-embolization syndrome).^11^

A range of materials are available for occluding the artery, including coils, glues, detachable plugs, thrombin, gelfoam, detachable balloons, and embolization copolymer.^11^^,^^17^^,^^18^ One of the principal ET techniques is coil embolization of saccular aneurysms and VAPAs, without impeding the flow through the principal artery.^14^ Deployment of a flow-modulating stent is also a treatment option for fusiform aneurysms with large diameters and little tortuosity.^14^ When the aneurysmal ring is narrow, another option is catheter-guided embolization with coils, detachable or otherwise. The most recent types of coils are of particular interest. They are detachable, are made from platinum (which is a more malleable material), and are helical in shape.^18^

In the case described here, after angiography and selective catheterization of the ICA, the artery was embolized with microcoils to seal the aneurysm sac then Nester^®^ platinum embolization coils (Cook Medical Inc., Indiana, United States) were released and controlled-release dacron fibers were deployed into the area of contrast leakage. The control angiography showed an absence of contrast leakage and preservation of the remaining arteries of the intestinal arch and the SMA. The coils described were chosen because of the compatibility they offer within the pseudoaneurysm sac, thereby averting any possible recanalization of the VAPA.^18^ In this case, endovascular treatment proved to be safe and satisfactory, with resolution of the patient’s symptoms and early discharge on the 3rd postoperative day.

## CONCLUSIONS

The case described illustrates the importance of early recognition and management of VAPAs with ET and coils, reducing morbidity and mortality in patients with acute abdomen secondary to hemorrhage of visceral arteries. Although this is a rare condition, similar situations to the one described are responsible for high lethality due to rupture and it is important to disseminate the technique and conduct in-depth studies of the subject. Long-term follow-up with larger numbers of patients will be needed to determine the final role of endoluminal treatment for visceral aneurysms and pseudoaneurysms.
