# Supplementary material for: Ruptured ileocolic artery pseudoaneurysm: case report
Source: J Vasc Bras. 2022 Jan 7;20:e20210163. doi: 10.1590/1677-5449.210163 (PMC8759586; doi:10.1590/1677-5449.210163)
Supplement: Translation

# Translation: Pseudoaneurisma roto de artéria ileocólica: relato de caso

**Authors:** Carolina Vasconcellos Sant’Anna, Felipe Garcia Kuhl, Alane Miranda Leite, Selma Regina de Oliveira Raymundo, André Rodrigo Miquelin, Vitória Acar, Vitor Brumato Fachini, Matheus Rafael Canuti

**Affiliations:** 1 Faculdade de Medicina de São José do Rio Preto – FAMERP, São José do Rio Preto, SP, Brasil.

**Keywords:** procedimento endovascular, falso aneurisma, artéria mesentérica superior

## Abstract

Os aneurismas de artérias viscerais (AAVs) e pseudoaneurismas de artérias viscerais (PAAVs) são condições raras e potencialmente letais quando rotos. Em geral, são encontrados incidentalmente na tomografia computadorizada de pacientes assintomáticos. Embora a cirurgia aberta convencional seja considerada atualmente o tratamento padrão ouro, a abordagem endovascular vem ganhando relevo por ser considerada um procedimento minimamente invasivo e com riscos cirúrgicos menores. Destaca-se, nessa abordagem, o uso de embolização por molas (*coils*) em AAVs e PAAVs saculares e implante de stent modulador de fluxo como alternativa de tratamento para aneurismas fusiformes. Apresentamos o caso de uma paciente de 51 anos com queixa de dor abdominal aguda, taquicardia e hipotensão, com evidência de sangramento abdominal em angiotomografia e diagnóstico de pseudoaneurisma de artéria ileocólica (AIC) roto. Ela foi submetida a tratamento endovascular precoce de embolização da AIC com sucesso, e houve melhora do quadro clínico.

## INTRODUÇÃO

Os aneurismas de artérias viscerais (AAVs) e pseudoaneurismas de artérias viscerais (PAAVs) são raros, com incidência entre 0,01 e 0,2%^1^. Com o avanço de técnicas em exames de imagem como a angiotomografia computadorizada (angioTC) de abdômen e o ultrassom Doppler (USD)^2^^,^^3^ para detecção de doenças intra-abdominais, juntamente com maior manipulação das vias biliares, a taxa de prevalência e o achado incidental dos aneurismas têm aumentado. Os AAVs são causados principalmente por hipertensão, aterosclerose, displasia e doenças do colágeno, enquanto os PAAVs estão associados a traumas, lesões iatrogênicas, inflamação ou infecções^4^. A artéria esplênica é a mais comumente afetada por aneurismas e pseudoaneurismas, seguida pela artéria hepática, tronco celíaco e artéria mesentérica inferior^5^.

Os PAAVs ocorrem mais frequentemente nas artérias gastroduodenal e mesentérica superior (AMS) se comparados a aneurismas verdadeiros, e os pseudoaneurismas rotos dessa artéria estão associados à taxa de mortalidade de 37%^6^.

Os aneurismas e pseudoaneurismas das artérias ileocólicas (AIC) são incomuns e geralmente assintomáticos, mas, quando sintomáticos, estão associados a sinais de rotura, tais como dor abdominal, sangramento intestinal baixo e hemorragia intra-abdominal ou sinais de choque hipovolêmico^7^. A cirurgia convencional é considerada padrão ouro no tratamento, porém o tratamento endovascular (TE) vem se destacando, principalmente na abordagem de pacientes com alto risco cirúrgico^8^^,^^9^.

Apresentamos o caso de uma paciente diagnosticada com PAAV de AIC roto submetida a TE com sucesso. Ressaltamos a importância do conhecimento dessa condição, que, embora rara e potencialmente fatal, pode apresentar um desfecho favorável se diagnosticada e tratada precocemente.

O estudo foi aprovado pelo Comitê de Ética e Pesquisa da nossa instituição (CAAE: 50239621.8.0000.5415, número do parecer consubstanciado: 4.919.534).

### Descrição do caso

Paciente do sexo feminino de 51 anos admitida em pronto atendimento com dor abdominal de forte intensidade em região superior há 2 dias e piora com o passar das horas, sem melhoria com uso de analgésicos. Na admissão, apresentava regular estado geral, palidez cutânea-mucosa, pele fria, pressão arterial de 125 x 59 mmHg, frequência cardíaca de 116 bpm, frequência respiratória de 17 ipm e temperatura corporal de 36,1º. No exame físico, o abdômen mostrava-se plano, com dor leve à palpação profunda na região superior, ausência de massa e/ou dor à descompressão. Foi solicitada angioTC abdominal, evidenciando-se volumoso hematoma intraperitoneal no mesentério na região do mesogastro de aproximadamente 800 cm^3^ e presença de líquido livre na cavidade abdominal em volume moderado. A AMS encontrava-se pérvia, e observou-se uma dilatação fusiforme em AIC com irregularidade de contornos e extravasamento de contraste ao longo do seu trajeto à direita (Figura 1A e 1B). A paciente negava doenças prévias e uso de medicamentos contínuos. Referiu ter sido submetida à colecistectomia videolaparoscópica há vários anos.

**Figura 1:** Angiotomografia de abdômen evidenciando: **(A)** artéria mesentérica superior apresentando dilatação fusiforme em artéria ileocólica com irregularidade de contornos e extravasamento de contraste; **(B)** volumoso hematoma intraperitoneal no mesentério e presença de líquido livre na cavidade abdominal.

Posteriormente, a paciente foi encaminhada ao setor de hemodinâmica, sendo realizada arteriografia por técnica de Seldinger com passagem de válvula hemostática 5Fr Performa^®^ (Merit Medical Systems, Inc., Utah, EUA) e posicionamento de cateter Cobra Performa^®^ (Merit Medical Systems, Inc., Utah, EUA), com injeção de contraste que identificou dilatação e extravasamento de contraste em AIC **(**Figura 2A e 2B). Foi também realizado cateterismo seletivo da AIC com microcateter Progreat (Terumo Corporation, Tóquio, Japão), e optou-se por embolização dessa artéria com liberação de micromolas Axium Prime™ (Medtronic, Dublin, Irlanda) de 2,5 x 6 cm em região final da dilatação e de micromolas Axium™ (Medtronic, Dublin, Irlanda) de 2,5 x 8 cm com selamento de *outflow*, seguido por liberação de molas de embolização Nester^®^ (Cook Medical Inc., Indiana, EUA) em área de extravasamento de contraste (5 molas 14 x 20 mm e 2 molas 14 x 18 mm), com selamento de *inflow* (Figura 3). Após o procedimento, a paciente apresentou melhora da dor e estabilidade hemodinâmica e recebeu alta hospitalar no 3° dia do pós-operatório (PO). No retorno de 3 meses, a paciente apresentava-se assintomática, e foi realizada angioTC controle, não sendo observados extravasamentos ou hematoma retroperitoneal (Figura 4A e 4B).

**Figura 2:** Angiografia de artérias abdominais mostrando: **(A)** pseudoaneurisma fusiforme em porção média da artéria ileocólica (seta preta); **(B)** extravasamento de contraste indicando rotura (seta vermelha).

**Figura 3:** Angiografia de controle após embolização da artéria ileocólica com micromolas evidenciando selamento do *outflow* e *inflow* e ausência de extravasamento (seta preta).

**Figura 4:** Angiotomografia controle após 3 meses do procedimento: **(A)** evidenciando as micromolas localizadas na artéria ileocólica e ausência de extravasamento; **(B)** imagem total do abdômen sem hematoma retroperitoneal.

### Discussão

Os AAVs e PAAVs são manifestações raras, com incidência entre 0,1 e 2%^1^, mas, apesar da baixa incidência, apresentam taxa de ruptura alta (25%) com morbimortalidade significante (70%)^5^^,^^10^. São causados por uma variedade de etiologias, e o diagnóstico inicial inclui USD e tomografia computadorizada (TC) com sensibilidade de 50% e 67%, respectivamente. A angiografia é o padrão ouro, pois permite a localização mais específica da lesão e confirmação do fluxo colateral, podendo ser usada para tratamento^11^. Os PAAVs de AMS são mais frequentemente causados por inflamação devido à pancreatite ou outras etiologias, além de poderem ser causados por traumas de origem iatrogênica^6^, desde cirurgia ou angiografia até acidentes ou traumas penetrantes^11^. A maior manipulação do trato biliar com técnicas percutâneas endoscópicas e implantes de cateter para quimioembolização intravascular resultaram em aumento da incidência de degeneração pseudoaneurismal de vasos viscerais, além de trauma arterial após tratamento laparoscópico de doenças intra e retroperitoneais^12^.

Com a disseminação das técnicas de imagem, como angioTC e angiorressonância magnética, houve um aumento no diagnóstico de AAVs e PAAVs e, muitas vezes, são achados incidentais em exames realizados para outras doenças abdominais^10^. A apresentação clínica dessas lesões é frequentemente vaga, e não há sinais claros alertando sobre a iminência de ruptura. Recomendam-se diagnóstico e manejo rápido em qualquer suspeita de PAAV em função de sua alta taxa de ruptura e hemorragia^11^. Pitton et al.^13^ constataram que PAAVs são mais propensos à ruptura que aneurismas verdadeiros (76,3% vs. 3,1%, respectivamente).

Houve, então, a necessidade de classificação para se decidir qual procedimento realizar: cirúrgico (aberto ou embolização por cateter) ou seguimento conservador^14^. A técnica aberta convencional que inclui ressecção com revascularização, ligadura ou ressecção final do órgão (em geral, esplenectomia) ainda permanece como padrão ouro de tratamento^8^^,^^15^. No entanto, pode, muitas vezes, interromper o fluxo sanguíneo da artéria principal e resultar em um quadro de isquemia intestinal em diferentes escalas^14^.

Tulsyan et al.^12^ demonstraram que a cirurgia convencional em locais de difícil acesso, com dificuldades técnicas e comorbidades associadas que não possibilitam a reconstrução vascular e em pacientes admitidos em situações críticas (sangramento agudo e rotura), pode potencializar a morbimortalidade. Eles evidenciaram taxa de sucesso de 96% na embolização endovascular com molas^12^.

Diante de algumas limitações na cirurgia aberta, o procedimento endovascular, por ser minimamente invasivo, tornou-se uma alternativa para pacientes que apresentavam maiores riscos cirúrgicos^8^^,^^9^^,^^16^. No caso de pacientes hemodinamicamente estáveis, o TE permanece a primeira opção para sangramento arterial conhecido e é considerado seguro e efetivo para tratar PAAVs. Uma vantagem para esse tipo de abordagem é permitir a individualização do tratamento para afecções diferentes, o que torna o método mais preciso e reduz as morbidades no PO. As complicações incluem ruptura do próprio saco, isquemia distal do saco pseudoaneurismático e possível fluxo arterial para o pseudoaneurisma após demorada reconstrução do fluxo (síndrome pós-embolização)^11^.

Uma variedade de materiais que promovem a oclusão da artéria está disponível, como molas, cola, plugues destacáveis, trombina, gelfoam, balões destacáveis e embolização de copolímero^11^^,^^17^^,^^18^. Uma das principais técnicas de TE é a embolização por molas em aneurismas e PAAVs saculares sem interrupção do fluxo da artéria principal^14^. O implante de stent modulador de fluxo também é uma alternativa de tratamento para aneurismas fusiformes com diâmetros largos e pequena tortuosidade^14^. Quando o anel aneurismático é estreito, uma opção é a embolização por molas guiadas por cateter, sendo destacáveis ou não. Entre os tipos de molas, ressaltam-se as mais recentes, que são destacáveis e constituídas de platina, um material mais maleável com forma helicoidal^18^.

No caso descrito aqui, depois da realização de angiografia e cateterização seletiva da AIC, foi feita embolização dessa artéria com micromolas para selamento do saco aneurismático e, na sequência, liberação de molas de embolização Nester^®^ (Cook Medical Inc., Indiana, EUA) de platina e fibras de dácron de liberação controlada na área de extravasamento de contraste. A angiografia de controle evidenciou ausência de extravasamento de contraste e preservação de demais artérias da arcada intestinal e da AMS. A escolha pelo uso das molas descritas ocorreu em razão da compatibilidade que elas proporcionam dentro do saco pseudoaneurismático, evitando, assim, uma possível recanalização do PAAV^18^. O tratamento endovascular nesse caso se mostrou satisfatório e seguro, com a resolução dos sintomas da paciente e alta precoce no 3º do PO.

## CONCLUSÃO

O caso descrito revela a importância do reconhecimento e abordagem precoce de PAAVs com TE e utilização de molas, com redução da morbimortalidade de pacientes com abdômen agudo hemorrágico de artérias viscerais. Embora seja uma condição rara, situações semelhantes à apresentada acarretam alta letalidade em função da rotura, sendo relevante a disseminação da técnica e aprofundamento de estudos acerca do tema. Acompanhamentos a longo prazo com maior número de pacientes serão necessários para elucidar o papel final da terapia endoluminal no tratamento dos aneurismas e pseudoaneurismas viscerais.
